# Effect of platelet-rich and platelet-poor plasma on peri-implant innervation in dog mandibles

**DOI:** 10.1186/s40729-019-0193-3

**Published:** 2019-12-04

**Authors:** Dandan Song, Yan Huang, Jeroen Van Dessel, Sohaib Shujaat, Kaan Orhan, Tim Vangansewinkel, Kathleen Van den Eynde, Ivo Lambrichts, Tania Roskams, Constantinus Politis, Reinhilde Jacobs

**Affiliations:** 1 OMFS IMPATH Research Group, Department of Imaging and Pathology, Faculty of Medicine, KU Leuven and Oral and Maxillofacial Surgery, University Hospitals Leuven, Campus Sint-Rafaël, Kapucijnenvoer 33, BE-3000 Leuven, Belgium; 2 State Key Laboratory of Oral Diseases, West China College of Stomatology, Sichuan University, Chengdu, China; 3 Department of Dentomaxillofacial Radiology, Faculty of Dentistry, University of Ankara, Ankara, Turkey; 4 Group of Morphology, Biomedical Research Institute, Hasselt University, Diepenbeek, Belgium; 5 Translational Cell & Tissue Research, Department of Imaging & Pathology, KU Leuven, Leuven, Belgium; 6 Department of Dental Medicine, Karolinska Institute, Stockholm, Sweden

**Keywords:** Platelet-rich plasma, Platelet-poor plasma, Dental implant, Histomorphometry, Myelinated nerve fibers, Innervation

## Abstract

**Background:**

Autologous plasma fractions, such as platelet-rich plasma (PRP) and platelet-poor plasma (PPP), contain growth factors that can enhance neural cell survival and are therefore likely to have the ability to promote nerve regeneration. The present study compared the effect of PRP and PPP application on myelinated nerve density and diameter in the peri-implant bone region. In addition, the effect of healing time on nerve regeneration was assessed.

**Materials and methods:**

Nine beagle dogs randomly received 54 dental implants in the bilateral mandible according to a split-mouth design. Each implant was randomly assigned to one of three implant protocols: delayed implant placement with delayed loading (DIP + DL) with local application of PRP, DIP + DL with local application of PPP and DIP + DL without any plasma additive. The animals were euthanized at 1, 3, and 6 months after loading (3 dogs per time point). Block biopsies were prepared for histomorphometry in the peri-implant bone within 500 μm around the implants.

**Results:**

Myelinated nerve fibers were identified in the trabecular bone and in the osteons near the implants surface. The nerve fibers in the PRP group (median ± IQR; 2.88 ± 1.55 μm) had a significantly (*p* < 0.05) greater diameter compared to the PPP (2.40 ± 0.91 μm) and control (2.11 ± 1.16 μm) group. The nerve diameter after 6 months healing (3.18 ± 1.58 μm) was significantly (*p* < 0.05) greater compared to 1 (2.08 ± 0.89 μm) and 3 (2.49 ± 1.22 μm) months. No significant difference was found for myelinated nerve density between groups and healing time.

**Conclusions:**

The present study showed that the healing time significantly influenced the diameter of the myelinated nerve fibers in peri-implant bone. PRP exerted a significant effect on the diameter of the myelinated nerve fibers as compared to PPP. Large-scale animal studies and longer follow-up periods are needed to confirm these findings and to verify whether platelet plasma can facilitate nerve regeneration process.

## Background

Dental implant surgery is one of the most widely accepted procedures for replacing missing dentition without harming the neighboring healthy teeth. The survival of the dental implant is dependent on successful osseointegration, defined as the direct structural and functional connection between vital bone and dental implant surface under a functional load. If optimal osseointegration is not achieved, biological failure and consequent implant loss can occur [1]. The residual alveolar ridge constantly undergoes modeling and remodeling following tooth extraction [2].

Maxillary and mandibular alveolar bone contains multiple nerve fibers, which are responsible for detecting mechanical loading-induced signals through the mechanosensitive cells knows as mechanoreceptors [3, 4]. These receptors are responsible for transmitting information from nerve endings on the magnitude, direction, and rate of occlusal load for sensory perception and neuromotor control. The mechanism of such receptors involves the transmission of sensitivity and pain when natural teeth are in hyperocclusion. Degeneration of the alveolar structure or periodontal ligaments (PDLs) can lead to the impairment of these receptors, hence effecting the neurosensory pathway [5]. As osseointegrated dental implants are highly susceptible to occlusal overload, the damaged receptors can directly result in the loss of fine exteroception [6]. The existing mechanoreceptors in the bone and periosteum play a significant role in tactile function following implant loading. The threshold level of active tactile force in implant-supported prostheses has been suggested to be lower than the complete denture but similar to that of the natural tooth [7–10].

Previous studies reported a partial restoration of peripheral sensory feedback pathway following implant placement. However, the underlying mechanism of this phenomenon remains unknown [11, 12]. Furthermore, neurophysiological and psychophysical evidence confirms peripheral receptor activation after active or passive loading of the implant. It is assumed that the latter could cause activation of endosseous and/or periosteal receptors in the peri-implant tissue [13]. In addition, histomorphological studies showed the presence of functional mechanoreceptors in the peri-implant region which might have been originally located in the periodontal ligament and neighboring periosteum [14, 15].

Myelinated nerve fibers are the most effective sensory signal transporters responsible for carrying these mechanoreceptors [16]. Several treatment strategies have been utilized for the regeneration of mechanoreceptors around osseointegrated dental implant which include, reconstruction of the peri-implant ligament [17], transplantation of Schwann cells (SCs) [18], injection of neuropeptides (e.g., calcitonin gene-related peptide-α) [19], and application of various implant placement and loading protocols [20]. Nevertheless, the clinical application of these therapies in implant surgery remains ambiguous.

Autologous plasma fractions, such as platelet-rich plasma (PRP) and platelet-poor plasma (PPP) have been utilized in dental implantology for stimulating new bone formation [21], angiogenesis [22], and peripheral nerve regeneration [23]. PRP is obtained by differential centrifugation of peripheral blood which divides the plasma, platelets, and leukocytes from red blood cells to form an upper plasma layer and intermediate buffy coat. The upper layer and superficial buffy coat are centrifuged for a second time to form the final PRP product, whereas, PPP is the residual plasma once the PRP is extracted [24]. The clinical potential of platelet concentrates depends on the number of platelets and the concentration of growth factors. Various growth factors, such as transforming growth factor-β (TGF-β), platelet-derived growth factor (PDGF), transforming growth factor (TGF), platelet factor interleukin (IL), vascular endothelial growth factor (VEGF), insulin-like growth factor (IGF), and basic fibroblast growth factor (bFGF), contained in the alpha-granules of platelets have been known be responsible for PRP-related effects. Although recent studies showed that PRP and PPP have comparable effects on bone [21] and blood vessel formation [22], no evidence is available comparing the effect of these two fractions on nerve innervation in the peri-implant bone. Therefore, the purpose of this study was to assess the effect of PRP and PPP on myelinated nerve density and diameter following delayed implant placement and delayed loading. In addition, the effect of healing time on peri-bone innervation was evaluated following 1, 3, and 6 months after loading.

## Materials and methods

### Study design

The study was approved by the Bioethics Committee of Sichuan University (reference number: WCCSIRB-D-2014-010). A split-mouth randomized study was designed in nine healthy male beagle dogs. The housing and feeding condition for all experimental dogs strictly followed the general program at Experimental Animal Center of Laboratory of Biotherapy.

### Sample size calculation

The minimum required sample size was calculated using the discrepancy in myelinated nerve diameter for delayed implant placement with delayed loading (1.07 ± 0.18 μm) and natural socket healing (1.23 ± 0.19 μm) obtained from a study with similar design [25]. An a priori power analysis in G*power 3.1 recommended minimum sample size of 18 peri-implant bone samples when assuming 80% power and α of 0.05 [26].

### Surgical procedure

**Fig. 1:**
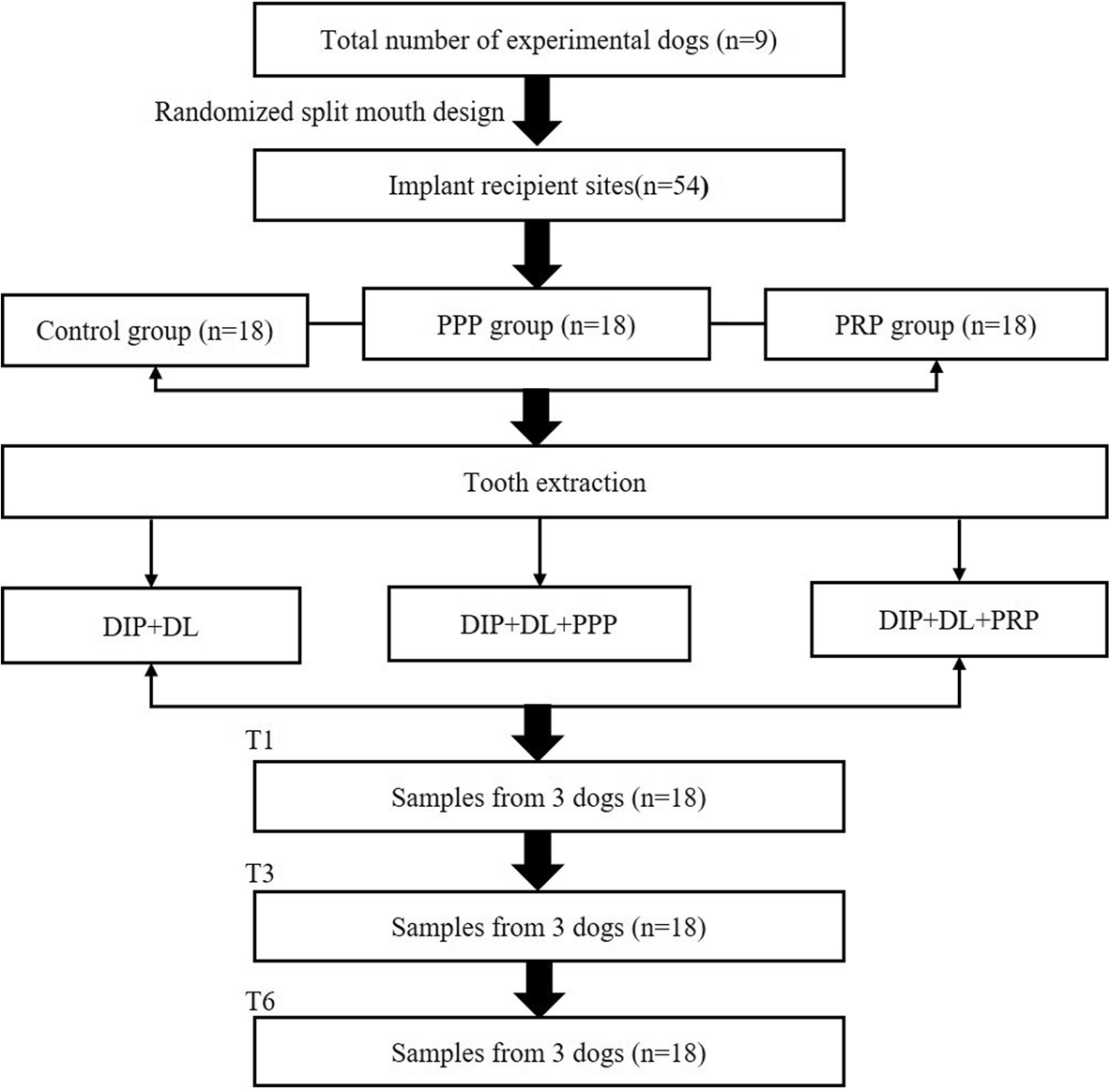
Flow chart of study design. DIP+DL, delayed implant placement and delayed loading. PPP, platelet-poor plasma; PRP, platelet-rich plasma; T1, 1 month healing time; T3, 3 months healing time; T6 6 months healing time

All animals (average weight 15.3 kg) received 1 week of prophylactic antibiotic therapy prior to and after surgery (Gentamycin Sulphate 300 mh, Tianjin Pharmaceutical, Tianjin, China). Bilateral extraction of mandibular third premolar, fourth premolar, and first molar was carried out. All surgeries were performed by the same oral and maxillofacial surgeon. After 1 month of natural healing, six dental implants without surface spiral burr (Beijing Leiden Biomaterial implant system, diameter 3.3 mm/length 8 mm) obtained from Leiden Biomaterial Limited Company, (Beijing, China) were placed bilaterally in the mandible of each dog. The surgical procedures were performed under general anesthesia with Sumianxin (0.1 ml/kg xylazine hydrochloride, Changchun Military Academy of Medical Sciences, Changchun, China) and local anesthesia (2–4 ml lidocaine 2% epinephrine, Tianjin Pharmaceutical Co. Ltd, Tianjin, China) was used at the surgical sites. The implant body part which was buried into alveolar bone was coated with a thin layer of plasma-sprayed hydroxyapatite (HA). Each implant was randomly assigned to one of the three implant protocols: delayed implant placement with delayed loading (DIP + DL) with a local application of PRP, DIP + DL with local application of PPP and DIP + DL without any plasma additive (Fig. 1). The surgeon was blinded to the allocation process during tooth extraction but aware of the exact position of implant placements. A crown was fabricated and attached to each implant at one month following surgery.

### Preparation and application of PPP and PRP

A double-centrifugation protocol was followed as suggested by Lee et al. [27]. Five milliliters of fresh whole blood was withdrawn from the foreleg vein of each dog and transferred into a sterile syringe, containing 1 ml of sodium citrate anticoagulant solution. The whole blood was first centrifuged (Allegra X 30R centrifuge, CA, USA) at 700 g for 8 min and then separated into four layers. Thereafter, supernatant plasma with a buffy coat was separated and transferred to a new centrifugation tube. It underwent a second centrifugation at 1600 g for 8 min. Finally, 1 ml of PPP and PRP were obtained separately and each implant in test groups was dipped in PPP and PRP solution prior to insertion in the alveolus.

### Occlusion restoration

The surgical condition and occlusion restoration were kept similar for all groups. All tissue-level implants were placed with their shoulders parallel to the level of marginal bone. Customized posts with resin crown (flowable resin composite under halogen light-curing unit for 20 s) were prepared with a resin cement (RelyX, Unicem, RX, 3M ESPE, St. Paul, USA). Occlusal contacts were kept edge-to-edge between implants and opposing natural teeth. The contacts were checked with an articulating paper (20 μm thick, Accufilm II, RX, 3M ESPE, St. Paul, USA).

### Animal sacrifice and histology

All dogs were healthy with clinically stable implants and normal surrounding soft tissue before sacrifice. Three dogs were randomly chosen utilizing a direct sampling technique at 1, 3, and 6 months’ time points (T1, T3, and T6). They were sacrificed using an overdose of xylazine hydrochloride (intravenous injection) and immediate perfusion of 4% paraformaldehyde and 0.0125% glutaraldehyde in 0.1 M phosphate buffer (pH 7.4). Specimen blocks were immersed in 0.5 mol/L ethylenediaminetetraacetic acid (EDTA) phosphate-buffered saline (pH 7.4) at 4 °C for 10 months, enabling easy removal of the implants using surgical forceps without damaging the samples. After dehydrated and fully infiltrated by paraffin, thin serial sections (~ 6 μm) were obtained by cutting in a buccal-lingual direction. All collected sections were then stained with Masson trichrome stain for histological analysis and detection of myelinated nerve fibers.

### Immunohistochemistry

The presence of myelinated nerve structures was confirmed with immunohistochemistry (IHC) by applying a labeled avidin-biotin method [28]. Sections were deparaffinized and microwaved using a 10-mm citrate buffer (pH 6.0). Thereafter, 0.5% H_2_O_2_ was applied to suppress endogenous peroxidase activity for reducing background staining. The unoccupied binding sites were blocked with 10% normal goat serum. Staining of the sections was carried out with primary antibody mouse monoclonal anti-neuropeptide Y (NPY, Santa Cruz Biotechnology, CA, USA, 1:50) followed by pretreatment with citrate (pH 6.0).

### Histomorphometric analysis

**Fig. 2:**
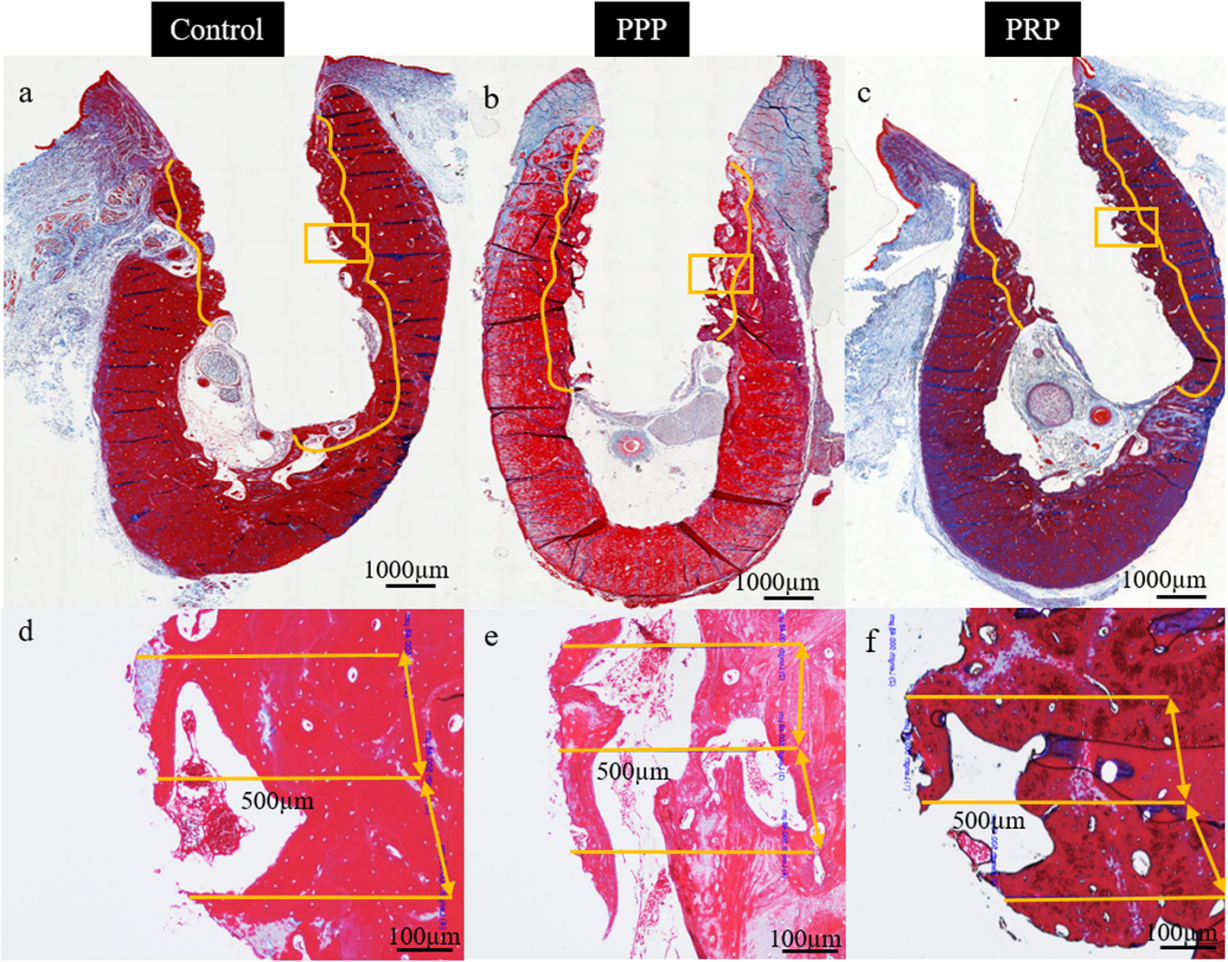
Region of interest selection 500 μm away from the implant surface on histological sections stained with Masson’s trichrome stain for control (**a**, **d**), platelet-poor plasma (**b**, **e**), platelet-rich plasma (**c**, **f**) group. All sections were after 6 months healing time and taken with a light microscope at × 10 (upper row) or × 40 magnification (bottom row)

Digitization and evaluation of three serial sections from every sample was performed with MiraxScan (Carl Zeiss, Göttingen, Germany). A single observer (DS), who was blinded to implant groups, evaluated the density (number of myelinated nerves/mm^2^) and outer diameter of myelinated fibers (μm) on a × 100 magnified image using Fiji software (LOCI in Madison, WI, USA). A region of interest (ROI) with a distance of 500 μm away from the implant surface was selected (Fig. 2), which is most likely to be influenced by the pressure from dental implant [29]. The partial fibers at borders of selected ROIs, myelinated nerve bundles and isolated axons in inferior alveolar nerve canal were excluded from evaluation.

### Statistical analysis

Normality in the distribution of data was assessed graphically and with the Shapiro-Wilk test. Non-parametric statistical tests were chosen by means of small sample size and non-linear data distribution. The descriptive analysis expressed data as median and interquartile range. The Kruskal-Wallis test was used to compare nerve density and shortest diameter values between implant protocols (control, PPP and PRP group) and time points (T1, T3, and T6). Dunn-Bonferroni corrected post hoc tests were used to explore significant interaction effects. A significance level α of 5% was considered for all tests. Statistical analysis was performed in SPSS (IBM, NY, USA).

## Results

**Fig. 3:**
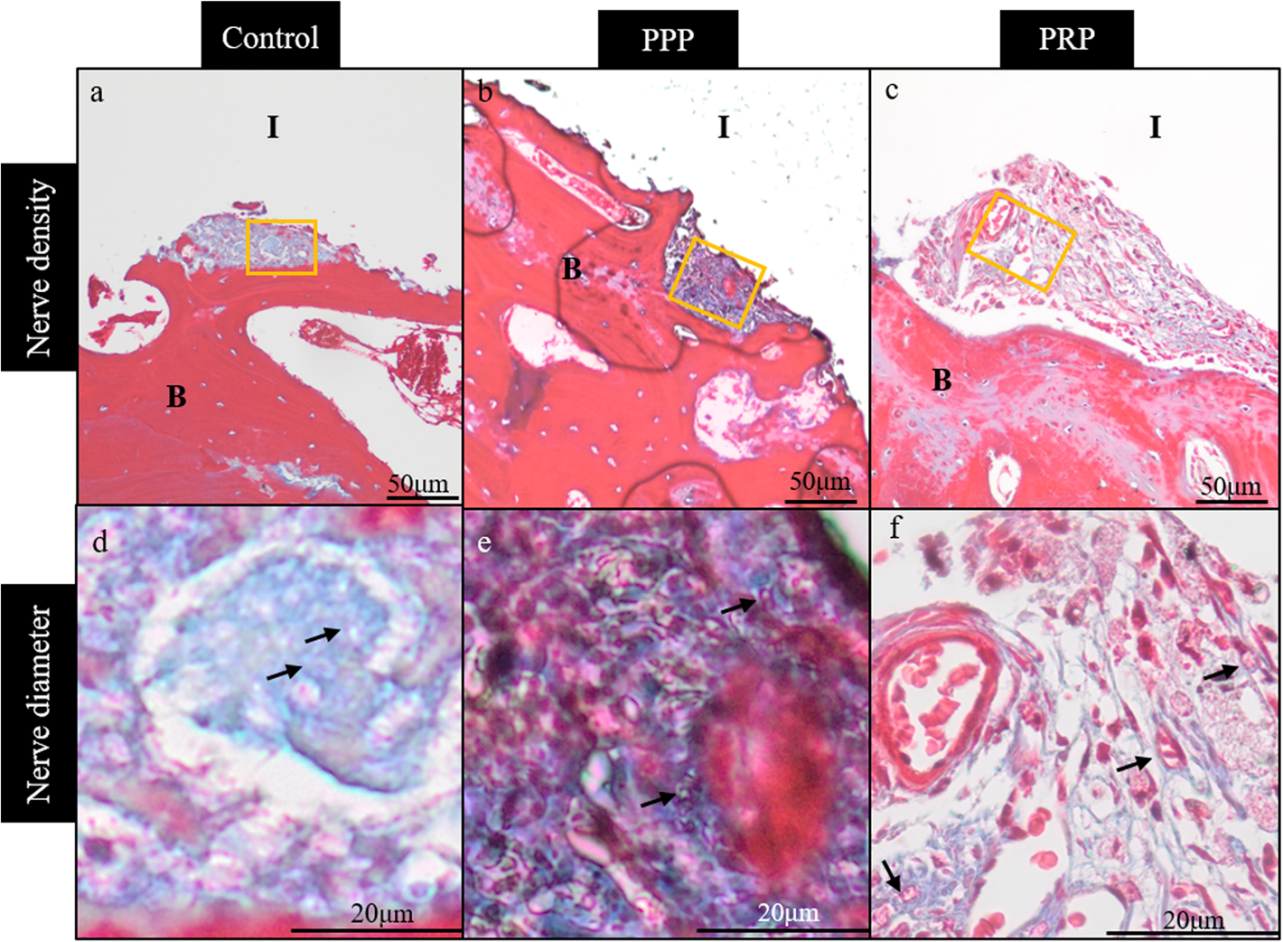
Histological sections stained with Massons’s trichrome stain for control (**a**, **d**), platelet-poor plasma (**b**, **e**), platelet-rich plasma (**c**, **f**) group near the implant surface. No difference was observed in the myelinated nerve density between the three groups (upper row). The nerve fiber diameter in the PRP group was greater than in the control and PPP group (see arrows, bottom row). All sections derive from 6 months healing time and taken with a light microscope at × 20 (upper row) or × 40 magnification (bottom row). I, implant; B, bone

**Fig. 4:**
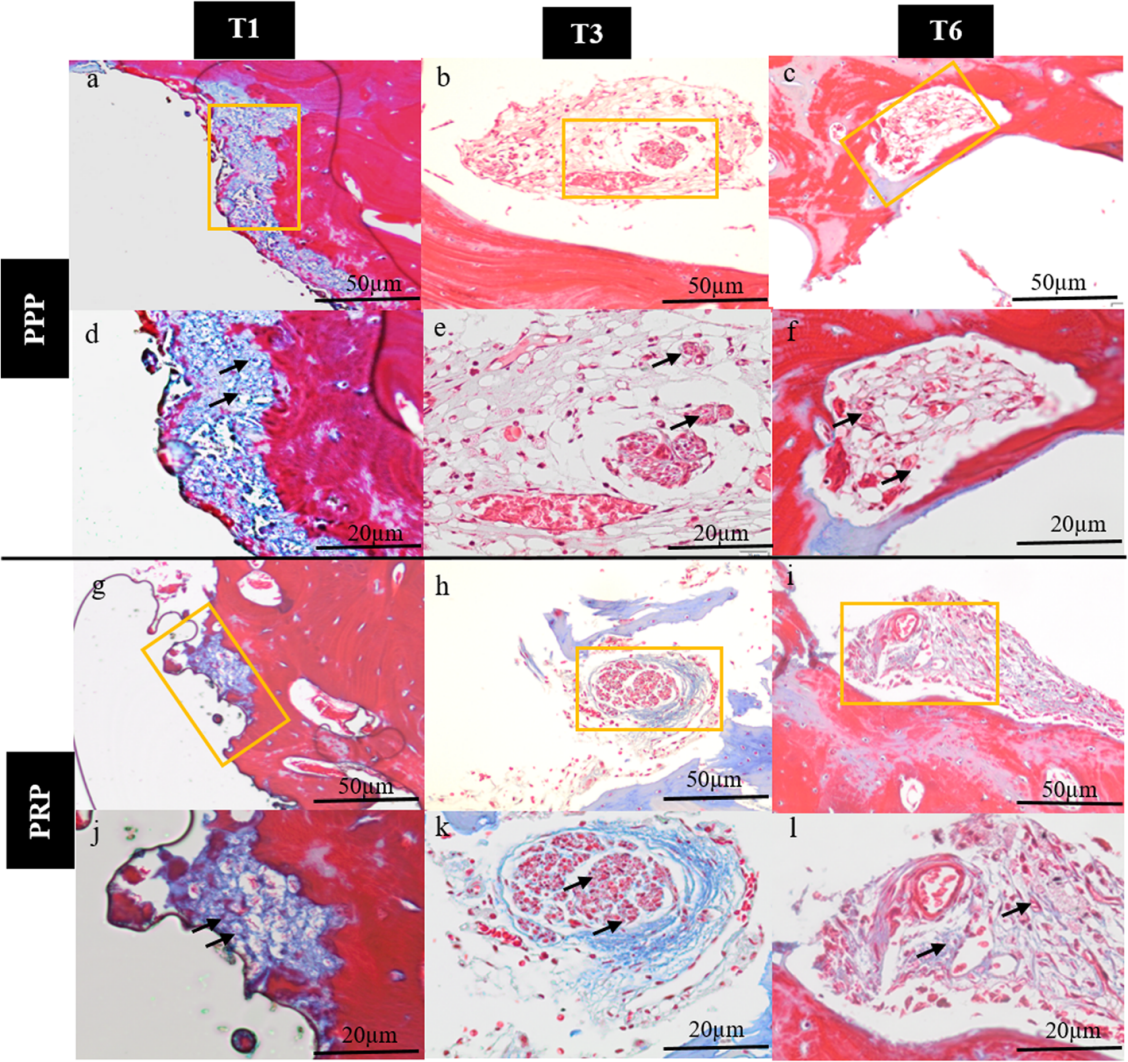
Histological sections stained with Massons’s trichrome stain for platelet-poor and platelet-rich plasma after 1 (T1), 3 (T3), and 6 months (T6) healing time. The myelinated nerve fibers diameter (see arrows) increased with longer healing times for PPP (**d**–**f**) and PRP (**j**–**l**), but no effect was seen on nerve density (**a**–**c** and **g**–**i**). All sections were acquired with a light microscope at × 20 or × 40 magnification

**Fig. 5:**
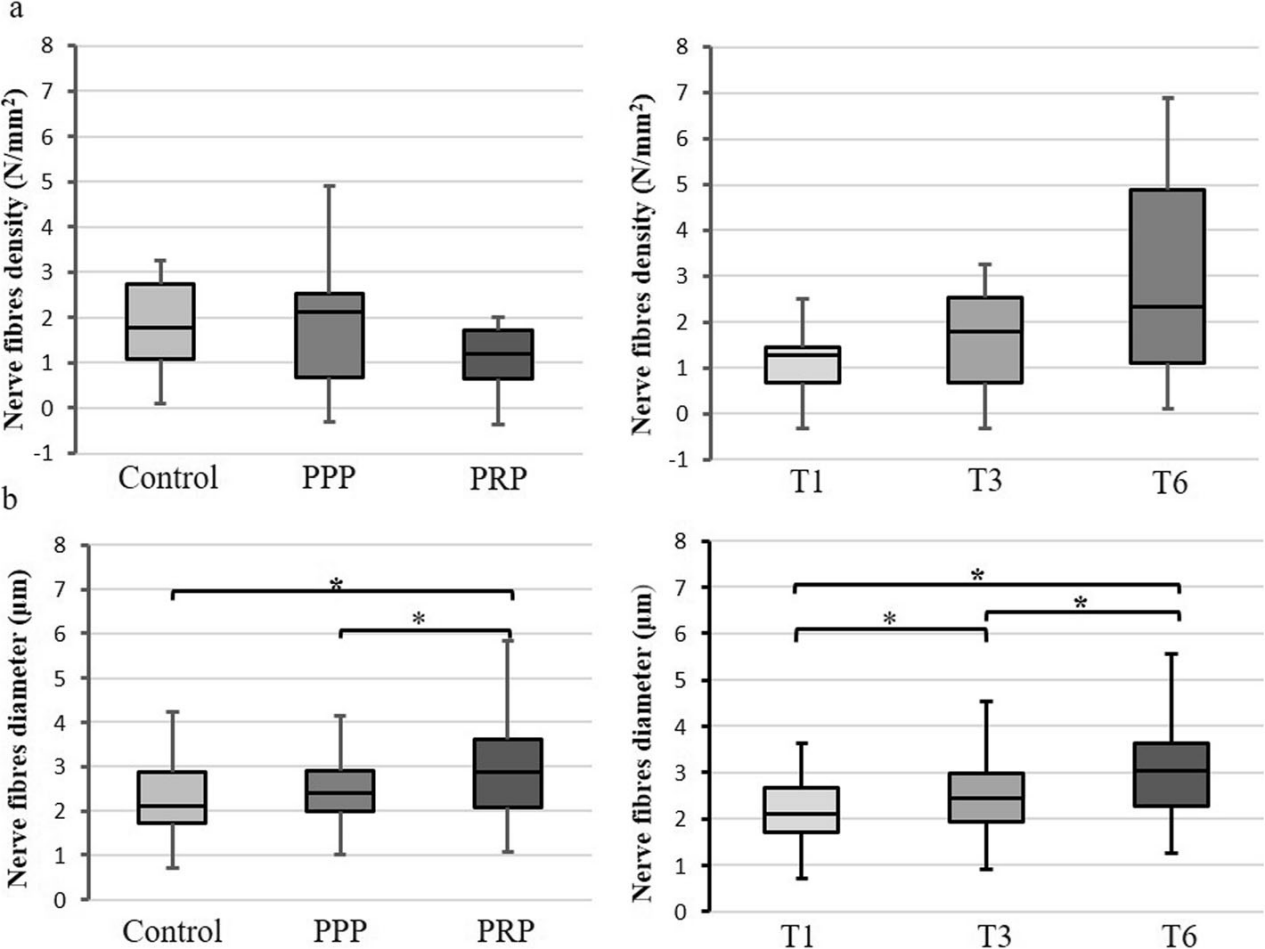
Box plots of nerve fiber density (**a**) and shortest diameter (**b**) for groups (control, platelet-poor, and platelet-rich plasma) and healing time (1, 3, and 6 months). The boundary the closest to 0 indicates the 25th percentile, a black line within the box marks the median, and the farthest from 0 indicates the 75th percentile. Whiskers above and below indicate the 10th and 90th percentile. No difference was observed in the myelinated nerve density, but a significant difference (*p* < 0.05 indicated by an asterisk) was found for the shortest nerve diameter between groups and healing time

All animals recovered well after implant placement and loading procedures without any clinical signs of infection or inflammation. All implants were clinically stable until euthanasia. Histological observation showed myelinated nerve fibers in the osteons near the implant surface and trabecular bone around the implant (Fig. 2). Nerve fibers were primarily dispersed perivascular and oriented according to the axis of the blood vessels. No difference was observed in the myelinated nerve density between the three groups (*p* = 0.58) and time points (*p* = 0.29) (Figs. 3 and 5). However, there was a significant (*p* < 0.001) difference between the three implant groups related to nerve diameter (Figs. 3 and 5). The nerve fiber diameter in the PRP group was greater than in the PPP (*p* = 0.02) and control (*p* < 0.001) group (Fig. 5). Overall, healing time significantly (*p* < 0.001) influenced myelinated nerve fiber diameter (Figs. 4 and 5). An increase in nerve diameter was observed at 6 months healing time compared to 1 (*p* < 0.001) and 3 (*p* = 0.002) months (Fig. 5).

## Discussion

The periodontal mechanoreceptors are an important component of the stomatognathic system. Tooth extraction leads to impairment of osseoperception by damaging these receptors [30]. The application of PRP has been demonstrated and proven to be beneficial for repairing damaged nerve fibers and receptors [31, 32]. Evidence suggests successful application of PRP for inducing nerve regeneration when the traumatic gaps of nerve structures are less than 3 cm long [33]. While the fact that the defects around dental implants are normally not as large as peripheral nerve defects might make the regeneration of peri-implant nerve fibers more feasible. Based on this fact, the present study was conducted to quantify the density and diameter of myelinated nerve fibers in peri-implant bone following local application of PRP and PPP. Moreover, the study focused on the clinical hypothesis that PRP contains numerous growth factors for promoting nerve growth.

The amount of growth factors in platelet plasma vary widely amongst different species. Van den Dolder et al. demonstrated in a comparative study that humans had a higher concentration of growth factors compared to other animal models [34]. Considering these differences amongst species, we applied regular double centrifugated protocol and separately transferred both the low concentration of PRP from the top layer and high concentration of PRP from the bottom layer into the implant bed.

In this study, the density and diameter of myelinated nerve fibers were examined in the region of 500 μm away from implant because the mechanoreceptors in this zone are considered to be easily activated by the loading pressure [29]. For minimizing the potential bias between experimental animals, a split-mouth design was applied with identical implant placement and the platelet plasma treatment protocols. The results showed a significant increase in diameter of myelinated nerve fibers after 3 and 6 months healing time. Furthermore, PRP exhibited a significant effect on the diameter of the myelinated nerve fibers as compared to PPP, with bigger diameter fibers observed in the PRP group. Wada et al. [35] reported an increase in the number of neurofilament protein (NFP)-positive nerve fibers after 4 months loading time.

When comparing myelinated nerve density amongst all three groups, a tendency was observed that PPP or PRP might help to improve regeneration of nerve fibers in peri-implant bone, more specifically 6 months after healing. Yet, this observation did not reach significance. This outcome could be explained based on short life of platelets (approximately 5–7 days) [36] and method of platelet plasma preparation and application. Literature reported that the concentration of PRP is dependent on its preparation process which can consecutively result in broad variability of growth factors [37, 38]. Graziani et. al reported that lower concentration of platelet plasma was better for enhancing cellular proliferation [38]. Cho et.al [39]. also found that PRPs’ biological effect on nerve fibers was dependent on its frequency of application and concentration which was not considered in our study. For further experiments, it could be advised to optimize the animal model and application protocol for PRP and use a larger subject sample to verify the present results.

Various surgical options have been applied for repairing injured peripheral nerves [40–42]; however, these strategies fail to provide a suitable regenerative micro-environment at a cellular and molecular level. To overcome this limitation, PRP has been applied as an adjuvant therapeutic strategy for promoting nerve regeneration and repair [43]. Recent evidence suggests a desirable effect of PRP related to regeneration of injured peripheral nerves and it has been successfully applied clinically for sensory and motor fibers repair of neuromuscular units [44]. In the same instance, PRP-coated dental implants have also shown to promote bone regeneration and accelerate soft tissue healing [40]. However, there is lack of evidence demonstrating the effect of PRP using inferior alveolar nerve and lingual nerve models. Based on our findings, we believe that local application of PRP in cases of iatrogenic inferior alveolar and lingual nerve damage during routine implant surgery may provide accelerated healing and regeneration of nerve fibers, thereby improving neurosensory recovery.

Despite the current study limitations, the present report provides for the first time an animal model to evaluate regeneration of injured nerve fibers in the proximity of dental implants. It is considered a step forward in understanding PRPs’ influence on implant rehabilitation surgery. Further studies should be performed to develop a standardized protocol for PRP preparation and application in the peri-implant region and assessing its effect on osseoperception.

## Conclusions

The present study showed that the healing time significantly influenced the diameter of the myelinated nerve fibers in the peri-implant bone. PRP exerted a significant effect on the diameter of the myelinated nerve fibers as compared to PPP. Large-scale animal studies and longer follow-up periods are needed to confirm these findings and to verify whether platelet plasma can facilitate nerve regeneration process.

## Data Availability

The datasets used and/or analyzed during the current study are available from the corresponding author on reasonable request.
